# A Methodological Assessment and Characterization of Genetically-Driven Variation in Three Human Phosphoproteomes

**DOI:** 10.1038/s41598-018-30587-3

**Published:** 2018-08-14

**Authors:** Brett W. Engelmann, Chiaowen Joyce Hsiao, John D. Blischak, Yannick Fourne, Zia Khan, Michael Ford, Yoav Gilad

**Affiliations:** 1 Department of Human Genetics, University of Chicago, Chicago, Illinois USA; 2 Department of Medicine, University of Chicago, Chicago, Illinois USA; 3 MS Bioworks, LLC, 3950 Varsity Drive, Ann Arbor, Michigan USA; 4 Present Address: AbbVie, North Chicago, Illinois USA; 5 Present Address: Genentech, South San Francisco, California, USA

## Abstract

Phosphorylation of proteins on serine, threonine, and tyrosine residues is a ubiquitous post-translational modification that plays a key part of essentially every cell signaling process. It is reasonable to assume that inter-individual variation in protein phosphorylation may underlie phenotypic differences, as has been observed for practically any other molecular regulatory phenotype. However, we do not know much about the extent of inter-individual variation in phosphorylation because it is quite challenging to perform a quantitative high throughput study to assess inter-individual variation in any post-translational modification. To test our ability to address this challenge with SILAC-based mass spectrometry, we quantified phosphorylation levels for three genotyped human cell lines within a nested experimental framework, and found that genetic background is the primary determinant of phosphoproteome variation. We uncovered multiple functional, biophysical, and genetic associations with germline driven phosphopeptide variation. Variants affecting protein levels or structure were among these associations, with the latter presenting, on average, a stronger effect. Interestingly, we found evidence that is consistent with a phosphopeptide variability buffering effect endowed from properties enriched within longer proteins. Because the small sample size in this ‘pilot’ study may limit the applicability of our genetic observations, we also undertook a thorough technical assessment of our experimental workflow to aid further efforts. Taken together, these results provide the foundation for future work to characterize inter-individual variation in post-translational modification levels and reveal novel insights into the nature of inter-individual variation in phosphorylation.

## Introduction

Protein phosphorylation is a ubiquitous mediator of information flow in essentially all cellular processes^1–4^, with a recent survey estimating that roughly 75% of the proteome can be phosphorylated^5^. Dysregulation of protein phosphorylation has long been recognized as a driver of disease^4,6–8^, and plays an important role in achieving and maintaining every ‘hallmark’ of cancer^9^. While the proteins involved and mechanistic details of the major phosphorylation mediated signal transduction pathways are largely known^2^, a growing body of research seeks to understand phosphorylation mediated information transfer as an integrated system using broad, quantitative, and unbiased surveys of the phosphoproteome combined with other ‘omic’ data^10–13^. Recent advances in liquid chromatography coupled to tandem mass spectrometry (LC-MS/MS) technology have enabled such surveys^5,14,15^, and multiple studies have reported the analysis of LC-MS/MS phosphoproteomic data together with genomic, transcriptomic, proteomic and metabolomic data^5,16–21^.

In particular, integrative phosphoproteomic-genomic studies have provided further evidence of the importance of phosphorylation in evolution and disease. Previous studies have combined genomic data with phosphoproteomic data to provide evidence that phosphorylation sites are conserved across species^22,23^, are under evolutionary constraint in humans^24^, and are over-represented in mutations that cause diseases in humans^24,25^. Phosphoproteomic data has also been combined with genomic and protein-binding specificity data to develop models that predict mutations likely to alter phosphorylation signaling in cancer^26^ or perturb specific kinases^27^. More recently, integrative phosphoproteomic-genomic studies have improved our understanding of how genetic alterations impact phosphorylation mediated signaling by combining LC-MS/MS derived quantitative phosphoproteomic and genomic data from the same samples. A recent integrative study identified signaling pathways that are differentially activated in breast cancer samples depending upon the mutation pattern of a frequently mutated gene^19^. In another example, phosphoproteomic data and exome sequence data collected from multiple ovarian cancer cell lines was used to assess the impact a subset of genetic variants have on a predicted phosphoprotein network state^16^. Despite this progress, we are not aware of any studies that have systematically characterized how genetic variation affects variation in phosphorylation levels across a set of commonly measured samples. Moreover, because many of the preceding *in vivo* studies were performed on cancer models, the contribution of heritable variation to naturally occurring inter-individual differences in protein phosphorylation levels remains unexplored.

Quantitative trait locus (QTL) mapping is a powerful approach to analyze inter-individual variation in phosphorylation levels. When QTL mapping is applied to molecular phenotypes, such as mRNA or protein expression levels, these are treated as quantitative traits. The goal of regulatory QTL mapping is to identify associations between inter-individual variation in the molecular phenotypes and the corresponding genotypes from multiple individuals^28^. Recent progress cataloging QTLs associated with various molecular phenotypes using high throughput approaches has been rapid^29–39^. Yet, to date, there have been no quantitative studies with an aim to characterize inter-individual variation in post-translational modification (PTM) levels. To begin addressing this gap, we performed a pilot study to assess the feasibility of QTL mapping PTM levels. We applied liquid chromatography coupled to tandem mass spectrometry (LC-MS/MS) to derive quantitative phosphoproteomes from three HapMap^40^ lymphoblastoid cell lines (LCLs) donated from Yoruba (Ibadan, Nigeria) individuals. Along with genomic information^41^, other quantitative datasets, including transcriptomic^30^ and proteomic^33^, have been previously collected from these LCLs. We leveraged these previous data sets and the quality of our phosphoproteomic data to explicitly estimate phosphopeptide variance arising from the genetic background. We found that the genetic background drives the majority of the observed variance, and uncovered many novel relationships between germline genetically-driven phosphorylation variation and diverse molecular annotations. We also included a power analysis with varying levels of increasing technical variance to aid the design of future studies.

## Results

### Nested deep quantitative phosphoproteome profiling

**Figure 1:**
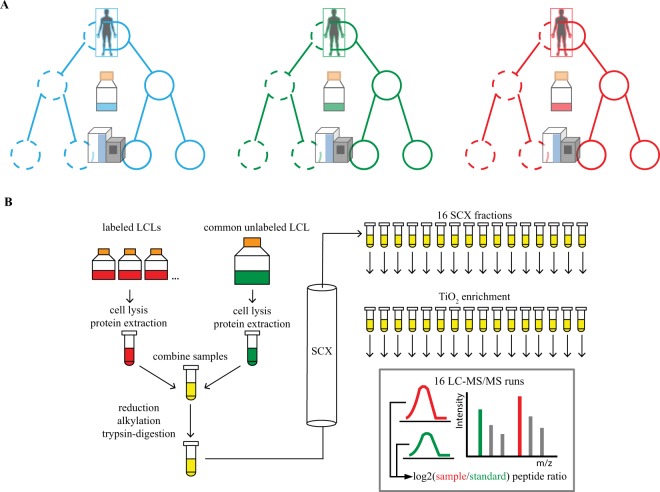
Nested SILAC-based phosphoproteomic analysis. (**A**) HapMap LCLs derived from three Yoruba males in Ibadan, Nigeria were repeatedly cultured and repeatedly subjected to a multistep mass spectrometry workflow. Dotted vs un-dotted circles represent the two different processing batches. (**B**) Protein extract from each stable isotope labeled sample is paired with an equal amount of extract from a common unlabeled cell line. This mixture is digested, separated into fractions via strong cation exchange (SCX) chromatography, and phosphopeptides are enriched with TiO_2_ resin and subjected to 1.5 hour HPLC runs on a Q-Exactive hybrid quadrupole-orbitrap mass spectrometer.

We applied a nested experimental design in order to characterize variation in protein phosphorylation between samples. We aimed to estimate the relative contributions from biological and technical sources to the observed variance in phosphopeptide quantification. We designed the study to specifically allow us to consider the contributions of genetic background, tissue culturing, and MS processing (Fig. 1A). We employed Stable Isotope Labeling by Amino Acids in Cell culture (SILAC)^42,43^ for relative quantitative comparisons of phosphopeptides using a common unlabeled reference LCL, and labeled sample LCLs (Fig. 1B). The phosphoproteome data set contains 192 1.5 hr gradient LC-MS/MS experiments on a Q-Exactive quadrupole orbitrap^44^, employing higher energy collisional dissociation to fragment peptides. Using this experimental approach, combined with the MaxQuant^45^ proteomic software suite and Andromeda^46^ search engine, we identified over 22,000 phosphopeptides from 5,143 unique protein groups at an FDR of 1% (Table 1, Supplemental Table 1). Ultimately, 17,774 phosphopeptides mapping to 4,584 protein groups produced spectra enabling confident localization of the site of phosphorylation and were assigned to SILAC pairs (‘Class 1’ quantifications, Table 1). The average mass error of the phosphoproteome is 0.1 ppm, roughly 53% of the phosphoproteome was commonly quantified in half of the samples, and peptides containing one phosphorylated serine residue are most common (Supplemental Fig. 1).

**Table 1:** Phosphopeptide level MS summary. All sites identified at an FDR of 1%.

Identified		Quantified		Class 1^†^ quantified		Class 1^†^ quantified in each* LCL		Class 1^†^ quantified in each* culture replicate		Class 1^†^ quantified and protein normalized in each* culture replicate	
Phospho peptides	Proteins	Phospho peptides	Proteins	Phospho peptides	Proteins	Phospho peptides	Proteins	Phospho peptides	Proteins	Phospho peptides	Proteins
22766	5143	21944	4845	17774	4584	11117	3514	4742	2073	3257	1181

^†^Refers to subset of phosphorylation sites with median localization probability of 0.99 and min 0.75.

*Refers to the intersection.

### Donor identity is the main biological source of phosphoproteome variation

As a first step of our analysis, we used normalized values (median-adjusted and quantile-normalized, see methods) to examine phosphopeptide variation prior to accounting for variation in protein expression levels. We applied principal component analysis (PCA) to this dataset and found that PC1 was associated with processing date, and PC2 was associated with donor identity (Supplemental Fig. 2A). These results indicated that a processing date batch effect is associated with substantial technical variation in our measurements. Thus, we applied the empirical Bayes approach ComBat^47^ to estimate and regress this batch effect from the data, applied PCA to the residuals, and visually determined that data across samples cluster by donor individual (Supplemental Fig. 2B). Following these results, the batch-corrected, normalized values were applied throughout our analysis.

**Figure 2:**
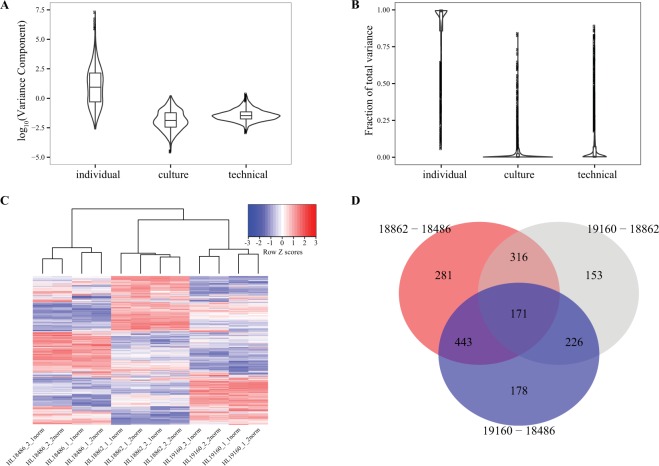
Genetically-driven phosphoproteomic variation. Violin plots of (**A**) absolute and (**B**) standardized phosphopeptide variance components derived from each layer of the hierarchical design after accounting for protein levels. (**C**) Heatmap of protein normalized phosphopeptide SILAC ratios. (**D**) Venn diagram of differential phosphorylation results across all three pairwise inter-individual comparisons (FDR 5%).

To explicitly account for the confounding effect of variation in protein expression levels, we assigned relative protein levels to each phosphopeptide using SILAC ratios collected from the same three LCLs. These SILAC ratios are derived from a previously reported MS dataset collected from 60 Yoruba LCLs, which employed the same reference sample as our study^33,48^. We processed these data with MaxQuant, yielding 3,885 identified and quantified protein groups in each of the three LCLs (the intersection) that we used in the current study (at a peptide and protein FDR of 1%; see methods, Supplemental Table 2). Because these SDS-PAGE protein expression levels were derived separately from the phosphopeptide data, we had to perform a separate normalization step and batch effect correction. We thus adopted a strategy that is commonly used in regulatory QTL studies to maximize the accuracy of molecular measurements. Specifically, to account for noise within the protein expression data we leveraged the available genotypes of the 60 LCLs to detect protein QTLs. Given that power to detect QTLs depends on the accuracy of the measurements, and that genotype distributions across *cis* regulatory loci are mostly uncorrelated, it is reasonable to assume that the protein data matrix that produces the most protein QTLs contains the most accurate protein estimates (indeed, an assumption shared by most regulatory QTL studies^30,33,49,50^). In order to identify this matrix, we iteratively applied PCA to the protein data and regressed unidentified confounders to maximize the number of protein QTLs identified across all 60 LCLs (see methods). Following the empirical correction of noise within the protein expression data, 1,181 protein groups were assigned to 3,257 phosphopeptides present in each of the three LCLs subjected to the phosphoproteomic work-up (Table 1). We note that although the proteome sampling is not as deep as recent studies, these protein groups span six orders of magnitude in expression and are therefore an unbiased sampling of the LCL proteome (Supplemental Fig. 3).

We used the corrected protein expression levels and the batch-effect corrected phosphopeptide values to estimate contributions to variance from the genetic background (the donor), culture replication (technical replication of the cell culture) and technical workup (protein sample processing and MS workflow). We fit a nested random effects model to each phosphopeptide with corrected protein expression levels as a covariate (see methods). We found that, for both absolute and relative phosphopeptide variance distributions, the genetic background dominates the observed variance (Fig. 2A,B). Next, we fit our nested random effects model two additional ways in order to assess the impact protein expression and our batch-effect correction approach had on our estimates. We analyzed the former by fitting our model with only normalized phosphopeptide measurements (Supplemental Fig. 4A,B) and the latter by fitting our model with both processing batch and corrected protein levels as fixed-effect covariates (Supplemental Fig. 4C,D). Accounting for protein levels resulted in a 36% increase in the median fraction of variance attributed to the donor while accounting for batch in the model resulted in a modest 2.5% decrease (Mann-Whitney tests; p values <= 4.23 × 10^−11^).

Next we considered differences in phosphorylation levels across the three LCLs. The study was not designed with a main aim to provide mechanistic insight into the specific pathways that drive inter-individual variation across LCL phosphoproteomes. Indeed, there is no specific stimulation response of interest and this is a small sample with which to attempt such analysis, just three individuals. Nevertheless, we hierarchically clustered the batch effect corrected and protein expression normalized phosphopeptide SILAC ratios and again found that the data cluster by donor (Fig. 2C). To focus our analysis on phosphorylation levels, we modeled the batch effect corrected phosphopeptide data while accounting for protein expression levels (see methods). Using this approach, we classified 48% (1577 of 3257) of the phosphopeptides as differentially phosphorylated between individuals (omnibus F test; FDR of 5%). We observed modest effect sizes (Supplemental Fig. 5), with 550 phosphopeptides having a fold-change greater than 2 in at least one comparison. We found a varied complement of differentially phosphorylated peptides and enriched gene ontology categories across inter-individual comparisons (Fig. 2D, Supplemental Tables 3–5). We also observed a variety of differentially phosphorylated phosphopeptide sequences, with only two kinase motifs enriched at an FDR of 5% (Supplemental Table 6) and no annotated kinases or single amino acid enriched at any sequence position across any inter-individual comparison (Supplemental Fig. 6). These findings demonstrate that there is an extensive amount of modestly varying phosphorylation across three non-stimulated LCLs derived from genetically different, albeit closely related donors.

### Characterization of genetically-driven differential phosphorylation

Understanding the nature of the genetic differences that putatively drive variation in protein phosphorylation between individuals is of fundamental interest and aids further experimentation. A key to this understanding is an assessment of the impact variants mapping to different functional categories have on inter-individual phosphopeptide variation. We undertook this assessment with an enrichment analysis of various annotations mapped to all phosphopeptides subjected to differential phosphorylation analysis (Supplemental Table 7).

To begin our assessment, we used the genomic sequence information available from all three donors^41^. Genetic variants impact phosphopeptide levels by altering protein expression or function. The former were previously captured within this system as protein QTLs (pQTLs)^33^, while the latter manifest via amino acid coding variants (non-synonymous SNPs and indels). We investigated all identified genetic differences between the three individuals in our study. In this case, though our sample size is small, the analysis relies on the large number of phosphopeptides we measured, and thus is not as underpowered as it may intuitively seem. Specifically, we are able to consider 19,002 coding variants affecting 8,656 unique genes and 181 pQTLs in these three individuals.

Using the p-values derived from the inter-individual F-tests we described above, we calculated (in a threshold independent fashion) the Spearman’s correlation between various genetic annotations and phosphopeptide variation^33^ (Table 2, see methods). A significant positive correlation between the presence of an annotation and phosphopeptide variability is indicative of an ‘enrichment’ of that annotation amongst proteins that contain phosphopeptides that are highly variable between individuals. Given that the expression level of a protein may impact phosphorylation levels in *cis* through enzyme-substrate titration, we hypothesized that proteins with annotated pQTLs would be enriched amongst those proteins that contain highly variable phosphopeptides. Consistent with this, we observed a significant enrichment of proteins with a pQTL (p = 0.03, Table 2). We also hypothesized that coding variation within a protein would correlate positively with phosphopeptide variability (in *cis*). Indeed, we found that proteins containing at least one non-synonymous variant were enriched (strongly, relative to proteins with annotated pQTLs) amongst those proteins that contain highly variable phosphopeptides (p = 6.48 × 10^−8^, Table 2).

**Table 2:** SNP categorical enrichment analysis.

Annotation (Protein)	N (phosphopeptides)	Background	p-value
At least one coding variant	3257	Phosphopeptides subjected to F-Test	6.48 × 10^−8^
pQTL	3257	Phosphopeptides subjected to F-Test	2.67 × 10^−2^
At least one variant within a Pfam domain	1332	Phosphopeptides within proteins that have at least one coding variant	9.83 × 10^−6^
At least one variant within a disordered region	1332	Phosphopeptides within proteins that have at least one coding variant	7.51 × 10^−1^
At least one PolyPhen HVAR Deleterious variant	1332	Phosphopeptides within proteins that have at least one coding variant	6.08 × 10^−6^
At least one PolyPhen HDIV Deleterious variant	1332	Phosphopeptides within proteins that have at least one coding variant	1.56 × 10^−3^
Phosphorylation regulation	1332	Phosphopeptides within proteins that have at least one coding variant	8.22 × 10^−1^
At least one variant within phosphorylation regulation domain	273	Phosphopeptides within proteins that have at least one coding variant in a Pfam domain	5.75 × 10^−1^

As a control for our approach we also tested whether the number of coding variants within a protein is correlated with inter-individual variability in phosphorylation levels. This should not be the case because the functional and biophysical context of a variant within a protein should have a greater impact on phosphopeptide variation than the overall number of variants within a protein. We tested this hypothesis by limiting the background set of phosphopeptides analyzed to those that are within proteins containing nonsynonymous variants. Within this background, we found that proteins with multiple coding variants are indeed not significantly enriched amongst those proteins that contain more variable phosphopeptides (>1 coding variant; n = 1,332, p = 0.11).

Thus, we proceeded by investigating the relationship between the context of genetic variants and phosphopeptide variation. We considered variant placement within the dichotomy of structured globular domains or disordered protein segments. While protein domains are the modules that largely impart protein function^51,52^, disordered regions contain most of the phosphopeptides observed to date^53^ (here 73%) and play critical roles in signal transduction and macromolecular assembly^54–56^. To supplement this dichotomy, we also categorized variants as likely or unlikely to impact protein function using PolyPhen-2^57^, which is an empirically trained prediction algorithm that considers multiple sequence and structure features. We limited the background set of phosphopeptides analyzed to those that are within proteins containing at least one nonsynonymous variant. Within this background, we found that both proteins with variants mapping to defined units of protein structure (domains) and proteins with variants likely to impact function (PolyPhen-2^57^ “deleterious” variants) are enriched amongst those that contain highly variable phosphopeptides (all p-values <=1.56 × 10^−3^; Table 2). However, we found that proteins with variants mapping to disordered regions are neither enriched nor depleted among those that contain highly variable phosphopeptides (p = 0.751, Table 2).

The function of a protein may also influence the likelihood that a variant impacts phosphorylation levels. Given their centrality within phosphorylation signaling networks, we investigated if proteins that regulate phosphorylation signaling are particularly impacted by genetic variation. Phosphorylation regulation proteins (PRPs) – kinases, phosphatases, and proteins containing non-catalytic phosphopeptide-recognition domains – alter phosphorylation levels in *cis* directly via catalysis or indirectly via spatial organization and subsequent catalysis by interacting proteins^1,58,59^. Notably, we did not find an enrichment of PRPs amongst proteins that contain highly variable phosphopeptides (nonsynonymous variant background, p = 0.82, Table 2). While a mutation within a PRP does not increase phosphopeptide variation relative to other proteins with mutations, it is possible that a targeted mutation within a kinase, phosphatase, or phosphopeptide-recognition domain is more likely to predispose such proteins toward increased phosphopeptide variability compared to mutations impacting other domains. To investigate this possibility, we limited the background set of phosphopeptides analyzed to those mapping to proteins that contain at least one variant in a domain. Within this background, we again did not find an enrichment of proteins with mutations in phosphopeptide regulation domains (p = 0.57, Table 2). Put together, these results indicate that the context of a mutation within a protein is the primary determinant of its ability to impact phosphopeptide levels in *cis*, regardless of any association that protein may have to phosphorylation regulation.

Lastly, we investigated the contextual impact variants may have on peptide-motif mediated interactions. Motif mediated protein-protein interactions and catalysis are important signal transduction mechanisms^60^. Binding of short linear peptides and phosphopeptides by PRPs directs specific catalysis and the formation of transient protein-protein interactions during signal transduction^1,60^. Motif defining amino acids are typically found within +/− 5 residues of the phosphorylation site^61–65^, with other residues extending beyond the motif also playing an important role to ensure interaction specificity^66^. While we were not able to obtain a large enough sampling of variants that disrupt annotated motifs (here only 5) to perform enrichment analysis, we did observe increased phosphopeptide variability as the distance between the phosphorylated site and the closest variant (in *cis*) decreased (R = −0.11; p = 4.37 × 10^−5^, Supplemental Fig. 7). This proximity effect is consistent with a signature of altered motif mediated protein-protein interactions.

### The putative functional impact of differences in phosphorylation

In order to further our understanding of differential phosphorylation we also analyzed the characteristics of the proteins and phosphopeptides associated with phosphorylation variation without explicit regard to coding variation. The characteristics we uncovered may not be specific to phosphopeptide variation driven by genetic differences, but generalizable to phosphopeptide differences driven by *eg* drug treatment. To do this, we carried out the same enrichment analysis approach outlined above but employed phosphopeptide or protein, rather than genetic, annotations (Supplemental Table 7).

Phosphorylation events may or may not result in changes to protein function^67,68^. Indeed, while phosphorylated sites are more conserved than non-phosphorylated sites^8,69,70^, this conservation is greatly increased when only considering phosphopeptides that have a known function^67,71^. Following batch-effect correction, the majority of the phosphopeptide variance observed in our study is derived from the genetic background rather than noise sources (Fig. 2A,B). Therefore, we hypothesized that phosphopeptides that map to regions of annotated function would be enriched amongst highly variable phosphopeptides. Indeed, we observed an enrichment of phosphopeptides that map to functional protein segments (domains or annotated motifs) amongst highly variable phosphopeptides (p = 6.50 × 10^−4^ (domains); p = 2.06 × 10^−7^ (motifs), Supplemental Table 8). Next, we asked whether phosphopeptides that map to phosphopeptide-regulation domains have altered variability relative to phosphopeptides that map to other domains. To test this, we limited the background set of phosphopeptides to those within domains. Within this background, we did not find an enrichment of phosphopeptides that map to phosphopeptide-regulation domains amongst highly variable phosphopeptides (p = 0.28, Supplemental Table 8). We also found no relationship between the functional association of a protein to phosphopeptide regulation and phosphopeptide variability (p = 0.06, n = 3257). These findings again support the notion that PRPs do not possess more variable phosphosites relative to other proteins and the context of a phosphopeptide within a protein is the primary indicator of its penchant for variability.

As noted above, phosphorylation sites are predominantly found within disordered regions between domains. Yet, we observed a depletion of phosphosites that reside within disordered segments amongst highly variable phosphopeptides (p = 6.00 × 10−3, Supplemental Table 8). This result was somewhat unexpected given that disordered regions are enriched in functional motifs^54,72^ and our observation above that phosphopeptides mapping to annotated motifs display (on average) increased variability relative to all phosphopeptides (Supplemental Table 8). We therefore hypothesized that the functional properties of proteins at the systems level may contribute to this observation. The relative importance of proteins within an interaction network may impact phosphopeptide variability in *cis*. For example, proteins with a high degree of connectivity (hubs) are more likely than proteins with a low degree of connectivity to be essential^73^. Protein hubs tend to be long, highly modified, and enriched in regions of structural disorder^74,75^.

Using externally derived annotations (see methods), we found that proteins with more interactions, more PTMs, and higher disordered residue content (captured by the percentage of disordered residues and the longest run-length of disordered residues within a protein) are depleted amongst those with highly variable phosphopeptides (all p-values <=0.03; Supplemental Table 9). Consistent with these annotation-derived observations, we also observed a depletion of highly phosphorylated proteins amongst those with highly variable phosphopeptides (p = 4.27 × 10^−6^, Supplemental Table 9). These observations could be driven in part by multiple mechanisms to direct specific protein-protein interactions such as the coordination between multiple PTMs, domains, and linear motifs that may be more common for longer proteins^3,54,55,76,77^. Indeed, we found longer proteins are depleted amongst those with highly variable phosphopeptides (p = 2.04 × 10^−5^, Supplemental Table 9).

The generally lower expression levels of longer proteins may also contribute to more “robust” phosphopeptide signaling due to mass action effects^78,79^. According to this hypothesis, lowly expressed proteins are less susceptible to PTMs resulting from promiscuous moderate affinity interactions. Consistent with this, we found lowly expressed proteins are depleted amongst those with highly variable phosphopeptides (p = 5.88 × 10^−6^, Supplemental Table 9). Taken together, these results indicate that the systems-level properties of a protein significantly impact the likelihood that phosphorylation levels will be altered in *cis*. Our results are consistent with a model where longer, lowly expressed, highly connected, and highly modified proteins are “buffered” from phosphorylation variation in *cis*.

## Discussion

### Technical assessment and recommendation for study design

In our study we applied an LC-MS/MS approach capable of deeply and reproducibly profiling the phosphoproteome to capture germline genetically-driven differential phosphorylation. We applied the same SILAC LCL standard^80^ that we used before^33,48^ in order to facilitate an integrative analysis. Critically, our application of a common SILAC standard allowed us to assess phosphorylation variation while controlling for variation in protein expression. This property of our study design also allowed us to address the systematic error produced from our multi-fraction technical workup (Fig. 1B). For our cell system and experimental approach, the magnitude of the donor associated variance component was on average much greater than either nested variance component, resulting in reasonable power to detect extensive differential phosphorylation between individuals (Fig. 2). As all our LCL samples were of similar passage and were cultured in practically identical conditions, we assume that environmental impact on differences between individuals is minimal. In other words, we assume that inter-individual variation in phosphorylation in our study stems from genetically-driven differences.

Given our inference that the genetic background drives most of the observed phosphopeptide variation, a less precise but also less laborious and time-consuming approach may actually be feasible for future studies. Recent reports of label-free LC-MS/MS phosphoproteomic approaches demonstrate greatly improved phosphoproteome sampling depth over previous label-free methods^14,15^. Label-free approaches have other benefits, such as an independence from the requirement to quantify peptides from a common standard. This independence would enable the quantification of phosphopeptides that are not found in a common standard but may be prevalent in other samples. Given that our results can inform future LC-MS/MS methodology choices, we performed simulations to assess the impact technical variance has on power (see methods). It is important to note that while this power analysis was performed without explicit regard to variation in DNA sequence, the effect sizes used are directly relevant for future phosphorylation QTL mapping studies where the aim is to identify relationships between coding variants and phosphorylation levels in *cis*. We found (Supplemental Fig. 8) that for our design (2 technical replicates), the power lost by increasing the technical variance up to 5-fold is almost completely compensated for by doubling the number of technical replicates. For our system (LCLs), this would be a welcome trade considering that this translates into less than half our currently required protein input and 1/8 of the instrument time (assuming 1 mg input and 1.5 hr gradients as in^14^).

### Functional associations with phosphopeptide variation

We uncovered multiple intriguing correlations between putatively genetically-driven phosphopeptide variation and functional annotations of polymorphisms and proteins. Of note is the apparently greater impact coding variants (especially those mapping to domains or those predicted to have functional consequences) have on phosphopeptide variation in *cis* relative to variants known to impact protein expression levels (Table 2). This observation implies that information relay via phosphorylation is more robust to variation in substrate protein levels than variation in substrate protein structure. This dichotomy may also portend a lack of concordance between pQTLs and phosQTLs (similar to that recently reported between eQTL and pQTL^33^) and relatively greater concordance between splicing QTLs and phosQTLs^39^. Future work employing additional samples is required to explore this property further.

We also uncovered novel aspects of phosphopeptide variation. For example, we observed an enrichment of phosphopeptides that map to functional protein segments (domains or annotated motifs) amongst highly variable phosphopeptides. We also consistently observed that PRPs such as kinases and phosphatases do not, on average, possess more variable phosphopeptides. From a systems perspective, we uncovered that the interaction count, PTM count, and disorder content of a protein correlate negatively with phosphopeptide variation in *cis*. Increased levels of these systems-relevant annotations are characteristic of longer, lowly expressed and tightly regulated proteins that are of amplified importance within interaction networks^73,78,79^. Intriguingly, the decreased variability of phosphopeptides mapping to such proteins may protect the cell from adopting unfavorable signaling states. We also observed increased inter-individual variability for phosphopeptides mapping to highly expressed proteins. This may result from random encounters with kinases and phosphatases and would therefore imply a lack of function^68^. Indeed, recent reports have found that highly expressed proteins are enriched in low stoichiometry phosphorylation sites with conservation rates similar to those of their non-phosphorylated counterparts^68^ and estimate that 80% of cellular ATP is consumed by only 20% of the (putatively functional) phosphorylation sites^5^. Future work will benefit from the application of these insights to prioritize phosphorylation events for further mechanistic characterization.

### Conclusions and Future Directions

We characterized inter-individual variation in PTM levels at substantial sampling depth with genotyped human cell lines. We provided evidence that variants affecting either protein structure or protein expression are associated with inter-individual phosphorylation variation. Our observations suggest that protein length, connectivity, and/or expression level may serve as a functional buffer against inter-individual phosphorylation variation. The generality of these results with respect to cell type, stimulation conditions, and sample size is currently unknown and requires further study. Lastly, our study demonstrates that current phosphoproteomic LC-MS/MS protocols are sufficient to capture germline driven PTM variation and provides a context for further technical development toward this end.

## Materials and Methods

### Cell culturing and SILAC labeling

Epstein-Barr virus (EBV)-transformed lymphoblastoid cell lines (LCLs) derived from Yoruba individuals in Ibadan, Nigeria (YRI from Coriell, NIGMS Human Genetic Cell Repository) were cultured under identical conditions of 37 °C and 5% CO_2_. Each of the three lines (GM18486, GM19160, and GM18862) were grown in Lys/Arg depleted RPMI and 15% dialyzed FBS supplemented with 2 mM L-glutamate, 100 IU/ml penicillin, 100 µg/ml streptomycin and L-^13^C_6_^15^N_4_-Arg (Arg-10) and L-^13^C_6_^15^N_2_-Lys (Lys-8) (Cambridge Isotopes, Andover, USA). Each line was cultured to ~200 × 10^6^ cells over at least six doublings. Culture replicates were awoken from the same frozen pellet and cultured in parallel. Label incorporation was verified by analyzing the protein lysate from the labeled LCLs alone by high-resolution LC-MS/MS. The internal (unlabeled) standard line (GM19238) was expanded to 20 × 10^9^ cells in roller bottles using RPMI media with 15% FBS and 2 mM L-glutamate by the Coriell Institute for Medical Research.

### Genotype data

The genotypes for the three YRI individuals were collected as part of the International HapMap Project^41^. Additional SNPs from the 1000 Genomes Phase1 integrated version 3 reference panel^81^ were imputed using IMPUTE2^82^, as previously described^35^. SnpEff ^83^ and SnpSift^84^ were used to identify all SNPs which had an effect on the amino acid sequence of a protein annotated in Ensembl GRCh37 release 75. PolyPhen2^57^ predictions were sourced from dbNSFP^85^ via SNPSift. A variant was included for analysis if it was observed in at least one allele in at least one of the LCLs except in the case where each LCL has the variant with the same genotype.

### Quantitative, high-resolution mass spectrometry

Cell pellets were washed twice with 500 µL 25 mM ammonium bicarbonate and centrifuged at 5000 × g for 2 minutes. Washes were discarded. Cell lysis was performed with 3 mL of urea lysis buffer (8 M urea, 50 mM Tris.HCl pH8, 100 mM NaCl) using 3 applications of a Qsonica Q125 sonic probe with a 30 second pulse and 80% amplitude. The cell lysate was centrifuged at 10,000 g for 10 minutes at 25 °C. The protein concentration of the cleared lysate was determined with a Qubit protein assay (Invitrogen). For each experiment 4.5 mg of light and 4.5 mg heavy protein were combined and digested with the following protocol: Reduction with 10 mM dithiothreitol at 25 °C for 30 minutes followed by alkylation with 20 mM iodoacetamide at 25 °C for 45 minutes. Proteins were digested with 200 µg sequencing grade trypsin (Promega) at 37 °C overnight. The final digest volume was 25 mL adjusted with 25 mM ammonium bicarbonate. The digestion was cooled to room temperature and terminated with 5 µL of formic acid. The digest was centrifuged at 10,000 g for 10 minutes. Peptides were desalted with 500 mg Sep-Pak (Waters) and dried using vacuum centrifugation in a SpeedVac. Dried peptides were dissolved in 7 mM KH_2_PO_4_, pH 2.65, 30% ACN and protein quantitation performed with a 280 nm protein assay. Peptides were fractionated on an Agilent 1100 equipped with a 500 uL sample loop operating at 2 mL/min, detector set at 220–280 nm wavelengths. 5 mg of peptide was loaded on polySULFOETHYL A, 4.6 mm ID × 200 mm length, 5 um particle size, 200 Å pore size (polyLC, from the Nest group). A total of 48 fractions were collected at 1 min intervals. In batches of 3, adjacent SCX fractions were pooled and processed by solid phase extraction (SPE) using a Waters SEP-PAK 50 mg C18 cartridge per the vendor protocol and dried overnight in a lyophilizer. Phosphopeptides were enriched using Titansphere TiO_2_ tips from GL sciences using the vendor protocol. Phosphopeptides were eluted from the tips using two eluents: 50 µL 5% NH_4_OH in water and 50 µL 5% Pyrrolidine in water. The eluents were combined and neutralized with 50% acetic acid and dried. Dry samples were reconstituted in 100 µL 0.1% trifluoroacetic (TFA) acid. Each enriched sample was desalted using a Stage Tip (ThermoFisher P/N SP301) per the vendor protocol. Peptides were dried and reconstituted in 70 µL of 0.1% TFA prior to analysis.

Half of each enriched sample was analyzed by nano LC-MS/MS with a Waters NanoAcquity HPLC system interfaced to a ThermoFisher Q Exactive mass spectrometer. Peptides were loaded on a trapping column and eluted over a 75 µm analytical column at 350 nL/min using a 2 hr reverse phase gradient; both columns were packed with Jupiter Proteo resin (Phenomenex). The injection volume was 30 µL. The mass spectrometer was operated in data-dependent mode, with the Orbitrap operating at 60,000 FWHM and 17,500 FWHM for MS and MS/MS respectively. The fifteen most abundant ions were selected for MS/MS.

### Computational analysis of mass spectrometry data

MS data was analyzed with MaxQuant^45^ 1.5.0.30 and the Adromeda^46^ search engine. Proteins were identified using a protein sequence database containing 35,585 consensus coding sequences (CCDS)^86^ translated from GRCh37/hg19 gene models using Ensembl release 75 annotations. Only translations of genes/transcripts of status ‘known’ or ‘novel’ and biotype ‘protein coding’ were used. Each sequence has a unique protein identifier (ENSP ID) that allows for mapping between gene and transcript IDs. Carbamidomethylation of cysteine was allowed as a fixed modification. N-terminal acetylation and oxidation of methionine as variable modifications were included for all searches, while phosphorylation of S/T/Y was included for the phosphorylation data. Up to three missed cleavages were allowed for phosphoproteomic data and two missed cleavages were allowed for proteomic data. A ‘first search’ tolerance of 40 ppm with a score threshold of 75 was used for time-dependent recalibration followed by a main search MS1 tolerance of 6 ppm and an MS2 tolerance of 20 ppm. The ‘re-quantify’ option was used to aid the assignment of isotopic patterns to labeling pairs. The ‘match between runs’ option was enabled to match identifications across samples using a matching time window of 42 seconds and an alignment time window of 30 min for phosphoproteomic data and 20 min for proteomic data. Peptide and protein false discovery rates were set to 1%. Quantitative analysis of phosphorylated peptides was limited to ‘class 1’ sites with median localization probability of 0.99 and minimum localization probability of 0.75. Protein group quantifications were taken as the median log_2_(sample/standard) ratio for all groups containing at least two independent unique or ‘razor’ peptide quantifications (including multiple measurements of the same peptide in different fractions) without a modified peptide counterpart. For the purposes of protein level normalization, a custom R script was used to assign phosphopeptides to protein groups based on the presence of the peptide within the sequences of the protein group proteins. In the rare cases where multiple protein groups contain proteins that match a phosphopeptide, the protein group with the most peptide identifications is assigned to the phoshpopeptide. For enrichment analyses, members of the protein group were parsed such that each member contained the given phosphopeptide sequence.

### Normalization and batch correction

Log-transformed phosphopeptide SILAC ratios were normalized in two steps. First, within each sample, we centered the log intensity ratios by subtracting the sample-specific median log intensity ratio. Second, we applied quantile normalization to account for between sample variation in log intensity ratios with the *limma*^87^ function ‘normalizeQuantiles’. We used ComBat^47^ to estimate and adjust for sample variation in the log intensity ratios attributable to processing date (with the *swamp*^88^ function ‘combat’).

### Variance component analysis

To assess the relative contributions of individual (donor identify), culture (technical replication of the cell culture), and technical workup (protein sample processing and MS workflow) variation to the observed logged SILAC intensity ratios of phosphopeptides, we fit a linear mixed model for each phosphopeptide on the quantile-normalized, batch effect-corrected data. The model estimates variance components due to the random effect of individual, culture, and technical workup as follows:

$${Y}_{ijk}={a}_{i}+{b}_{j(i)}+{\beta }_{i}^{protein}\,\ast \,protei{n}_{i}+{\varepsilon }_{k(ij)}$$

where ${Y}_{ijk}$ denotes the observed logged intensity ratio of individual *i*, derived from the *k*-th technical workup of the *j*-th culture replicate, with *i* = GM18486, GM19160, GM18862, *j* = 1, 2, and *k* = 1, 2. ${a}_{i}$ denotes random effects of individual, ${b}_{j(i)}$ estimates the random effect of *j*-th culture replicate for individual *i*, and ${\varepsilon }_{k(ij)}$ denotes the random effect of *k*-th technical workup in *j*-th culture replicate for individual *i*. Protein expression levels ${\beta }_{i}^{protein}$ in individual *i* are included as fixed covariates to account for the confounding effect of variation in protein expression levels. The protein expression measurements for each phosphopeptide were derived from SILAC protein expression ratios previously reported in an MS dataset collected from 60 Yoruba LCLs^48^. These protein data were processed as described under our Results section entitled *Characterization of genetically-driven differential phosphorylation*. The random effects of individual sample ${a}_{i}$, culture replicate ${b}_{j(i)}$, and technical workup ${\varepsilon }_{k(ij)}$ are assumed to be independent and follow normal distributions with zero mean and variance components ${\sigma }_{a}^{2},\,{\sigma }_{b}^{2},\,{\sigma }_{\varepsilon }^{2}$, respectively. The R package *MCMCglmm*^89^ was used to estimate the variance components associated with the random effects. A similar analysis was performed for Supplemental Fig. 4 using normalized phosphopeptide data and processing date batch as a covariate.

### Differential phosphorylation analysis

To quantify individual differences for each phosphopeptide in the observed logged SILAC intensity ratios, we fit a linear mixed effect model on the quantile-normalized, batch effect-corrected data: including individual as a fixed effect, culture replication as a random effect and logged protein SILAC intensity ratios as a fixed covariate. Our approach is based on *limma* – a popular linear-model based approach for differential abundance analysis in genome-wide expression studies. In our model, we also include weights for each phosphopeptide to account for the relationship between the model residuals of SILAC intensity ratios and the average log intensity of the phosphopeptides. Specifically, the model residuals of SILAC intensity ratios are negatively correlated with log intensity of the phosphopeptides. We computed observation-level weights of the model residuals using the *voom* approach^90^. A similar approach was used in previous work on modeling differential abundance in label-free LC-MS/MS proteomic experiments^91^.

Furthermore, we explicitly accounted for noise in the protein level estimates by a commonly used strategy for maximizing the accuracy of molecular measurements in regulatory eQTL studies^33^ (see Results section for more details). Briefly, we serially regressed PCs from the full protein data matrix derived from 60 LCLs^33^ and identified pQTLs from the resulting residual matrix. The residual matrix with the first 13 PCs regressed produced the maximum pQTL count and was therefore employed here. The Benjamini and Hochberg^92^ procedure was used to compute false discovery rates (FDR) via the ‘p.adjust’ function from the R package *stats*^93^. Significant individual variation in the phosphopeptide intensity ratio was identified at 5% FDR.

### Power analysis

We investigated the power of our differential phosphorylation analysis to detect significant individual variation. Specifically, we estimated the number of technical replicates required (per culture replicate) to reach 80% power, given varying levels of sampling noise from technical workup. The power calculation proceeds as follows:

1. Identify phosphopeptides with significant individual variation, and among these, choose the one phosphopeptide with the largest p-value and compute its effect size (F-statistic).
2. Based on the choice of phosphopeptide, extract all parameter estimates in the differential phosphorylation analysis, including the effect sizes of individual variation (F-statistic) and protein expression covariate, and the variance components of culture replicate and technical workup.
3. Simulate 1,000 peptides under the model assumptions of differential phosphorylation analysis. Use parameter estimates from Step 2. Fix the effect sizes of individual variation and protein expression levels, and the variance component of culture replicate. Vary the number of technical replicates and variance components of technical workup.
4. Given the settings in Step 2, we compute power as the probability of detecting significant individual variation in each simulation at FDR 5%.

### Protein, domain and phosphosite annotation

Pfam^94^ domain assignment and boundary definition was accomplished using InterProScan^95^ and Ensembl 75 CCDS FASTAs. Kinases, phosphatases, and modular phosphopeptide binding domains with the following Pfam family identifiers were considered functionally relevant for phosphorylation mediated signaling: PF00498, PF01846, PF03166, PF10401, PF00244, PF00533, PF00400, PF00659, PF00397, PF00782, PF06602, PF04273, PF14566, PF14671, PF04179, PF05706, PF00069, PF01636, PF03109, PF03881, PF06293, PF01163, PF01633, PF10707, PF06176, PF02958, PF04655, PF10009, PF12260, PF16474, PF07914, PF14531, PF06734, PF05445, PF07387. Gene Ontology^96^ IDs were sourced using biomaRt^97^. PTM site datasets and kinases annotated as phosphorylating specific sites were sourced from PhosphoSitePlus^98^ on 9/8/15. Human physical protein-protein interaction data was sourced from BioGRID^99^ v3.4.127 on 8/25/15. Eukaryotic Linear Motif (ELM)^60^ instances were sourced on 10/7/15 and mapped to proteins and phosphosites using custom R scripts. Kinase motifs were sourced from the Human Protein Reference Database (HPRD)^100^ via MaxQuant’s Perseus module. Protein level disorder was predicted using the RAPID^101^ algorithm and webserver on 8/28/15. Amino acid disorder was predicted with IUPred^102^. Scores ≥ 0.5 were considered disordered.

### Enrichment analyses

We assessed gene ontology enrichment on phosphopeptides categorized as differentially expressed (5% FDR) across each contrast using one-sided Fisher’s exact tests. Adjusted p-values were derived using the approach of Benjamini and Hochberg^92^ via the ‘p.adjust’ function from the ‘stats’ R package^93^. Distributions of nominal p-values derived from *limma* omnibus F-tests were used to assess the enrichment of annotations as outlined previously^33^. For each test, we calculated Spearman’s correlations between a vector of negative log-transformed *limma* F-test p-values and a binary vector designating assignment of the protein containing the phosphopeptide, or the phosphopeptide itself to an annotation. The p-value for the Spearman’s correlation was computed with the R function ‘cor.test’ with the option ‘exact = FALSE’. Amino acid position specific enrichments were produced with ‘pLogo’^103^.

### Code and data availability

The custom R^93^ scripts used in this study are available from https://github.com/bengalengel/Phospilot. The mass spectrometry proteomics data have been deposited to the ProteomeXchange Consortium via the PRIDE^104^ partner repository with the dataset identifier PXD008002. SILAC protein estimates are available from proteomeXchange; identifier PXD001406. pQTL data are available from http://www.sciencemag.org/content/suppl/2014/12/17/science.1260793.DC1/1260793_DatafileS1.xlsx. Genotype data are available from http://eqtl.uchicago.edu/jointLCL/genotypesYRI.gen.txt.gz.

## Electronic supplementary material

Supplemental Figures

Supplemental Table 1

Supplemental Table 2

Supplemental Table 3

Supplemental Table 4

Supplemental Table 5

Supplemental Table 6

Supplemental Table 7

Supplemental Table 8

Supplemental Table 9
