# Supplementary material for: A Methodological Assessment and Characterization of Genetically-Driven Variation in Three Human Phosphoproteomes
Source: Sci Rep. 2018 Aug 14;8:12106. doi: 10.1038/s41598-018-30587-3 (PMC6092387; doi:10.1038/s41598-018-30587-3)
Supplement: Supplementary file 1 — Supplemental Figures [file 41598_2018_30587_MOESM1_ESM.pdf]

Supplementary Information

A Methodological Assessment and Characterization of Genetically-Driven Variation in Three Human Phosphoproteomes

Brett W. Engelmann[1,4*†], Chiaowen Joyce Hsiao[1†], John D. Blischak[1], Yannick Fourne[1], Zia Khan[1,5], Michael Ford[3], Yoav Gilad[1,2*]

[1]Department of Human Genetics, University of Chicago, Chicago, Illinois, USA
[2]Department of Medicine, University of Chicago, Chicago, Illinois, USA
[3]MS Bioworks, LLC, 3950 Varsity Drive, Ann Arbor, Michigan, USA
[4]Current Address: AbbVie, North Chicago, Illinois, USA
[5]Current Address: Genentech, South San Francisco, California, USA

[*]Correspondence to: bengelmann@uchicago.edu (B.W.E); gilad@uchicago.edu (Y.G.)
[†]Equal Contribution

Supplemental Figure 1. Phosphoproteome summary statistics. (A) Overlapping class one phosphosites across all samples. **(B)** Number of phosphosites per protein. **(C)** Mass error of all identified phosphopeptides (median = .1 parts per million). **(D)** Class one phosphopeptide amino acid distribution. **(E)** Distribution of class one phosphopeptides with one, two, or three phosphorylated sites. **(F)** Site level overlap of singly, doubly, and triply phosphorylated class one phosphopeptides.

Supplemental Figure 2. Batch effect identification and post-correction exploratory data analysis. (A) PCA of SILAC phosphopeptide ratios. **(B)** PCA of SILAC phosphopeptide ratios following batch effect correction with ComBat.

Supplemental Figure 3. Protein expression dynamic range. Intensity-based absolute quantification (iBAQ) values for the 1,181 protein groups assigned to 3,257 phosphopeptides plotted against their rank.

Supplemental Figure 4. Phosphopeptide variance component estimates. Violin plots of **(A)** absolute and **(B)** relative phosphopeptide variance components without accounting for protein expression levels. Violin plots of **(C)** absolute and **(D)** relative phosphopeptide variance components after accounting for processing date and protein expression levels. Relative variance components were calculated by dividing the absolute value by the sum of all three variance components.

Supplemental Figure 5. Volcano plots for each inter-individual comparison. Phosphopeptides categorized as differentially expressed at an FDR of 5% are highlighted in red.

Supplemental Figure 6. Amino acid probability logos for differentially expressed phosphopeptides. (A) – (F). Logos are presented for each inter-individual comparison and fold change direction. The position-specific amino acid enrichment odds are calculated relative to the complement of phosphoserine peptides subjected to differential phosphorylation analysis.

Supplemental Figure 7. Relationship between SNP proximity and phosphopeptide variability. The primary protein structure 'distance' between the nearest non-synonymous variant (in *cis*) to each phosphopeptide was regressed against nominal F-test P-values. Pearson's correlation coefficient and P-value inset. A pseudocount of 1 was added to avoid undefined values.

Supplemental Figure 8. Relationship between differential phosphorylation power and technical replication. Power to detect differential phosphorylation is shown relative to multiples of observed technical variance. Lines are specific to the number of technical replicates.

Figure S1

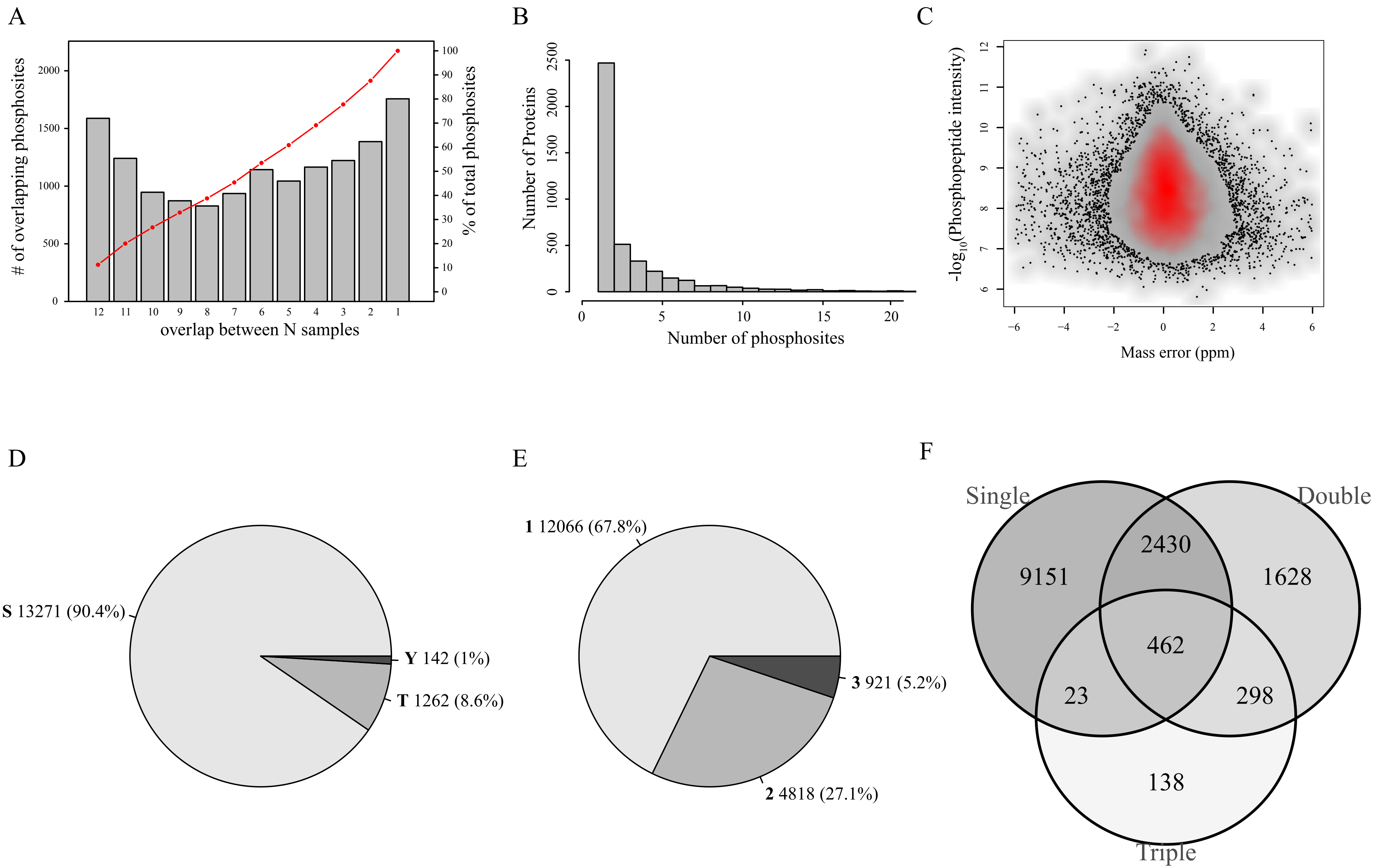
A
# of overlapping phosphosites
% of total phosphosites
overlap between N samples
B
Number of Proteins
Number of phosphosites
C
$-\log_{10}$(Phosphopeptide intensity)
Mass error (ppm)
D
S 13271 (90.4%)
Y 142 (1%)
T 1262 (8.6%)
E
1 12066 (67.8%)
3 921 (5.2%)
2 4818 (27.1%)
F
Single
Double
Triple
9151
2430
1628
462
23
298
138

Figure S2

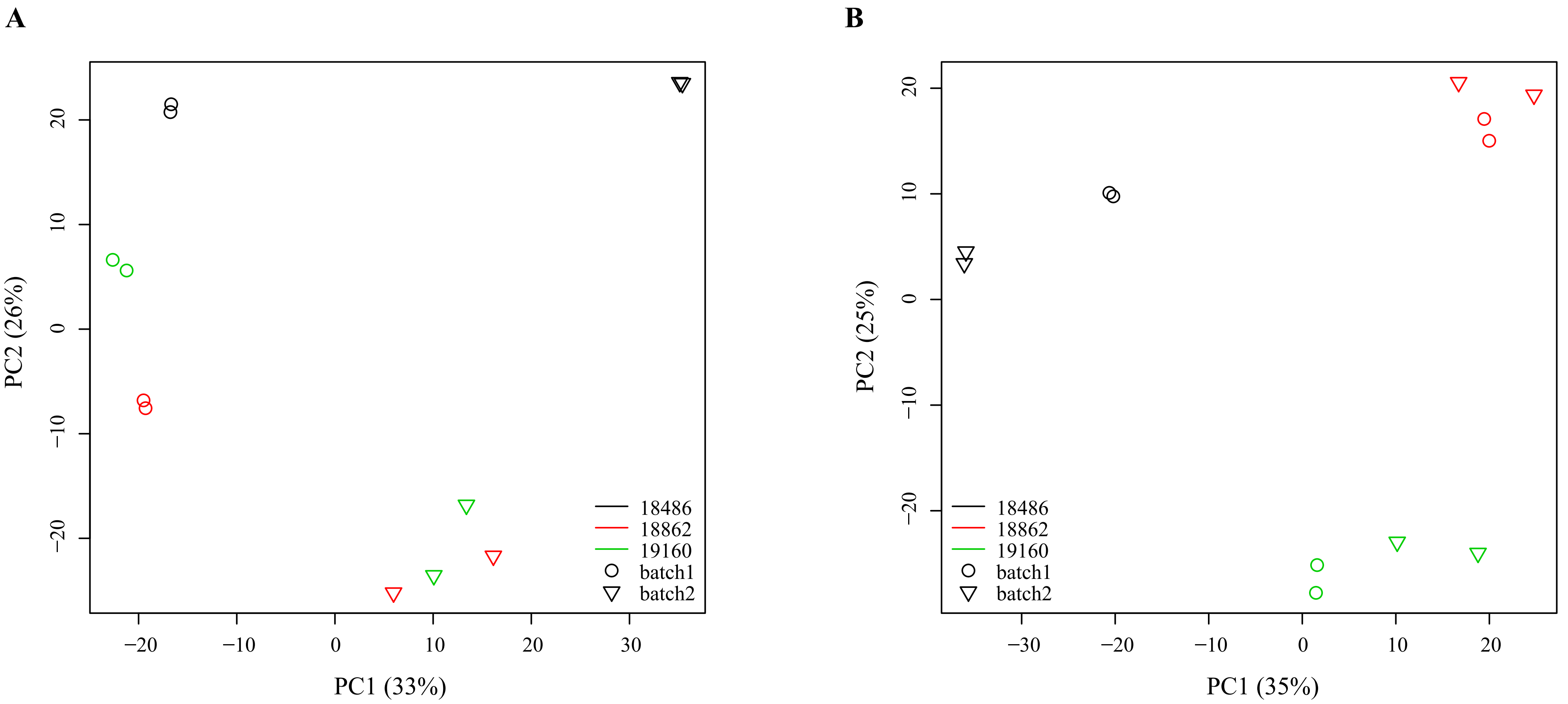
A
PC2 (26%)
PC1 (33%)
−20
−10
0
10
20
30
18486
18862
19160
batch1
batch2
B
PC2 (25%)
PC1 (35%)
−30
−20
−10
0
10
20
18486
18862
19160
batch1
batch2

Figure S3

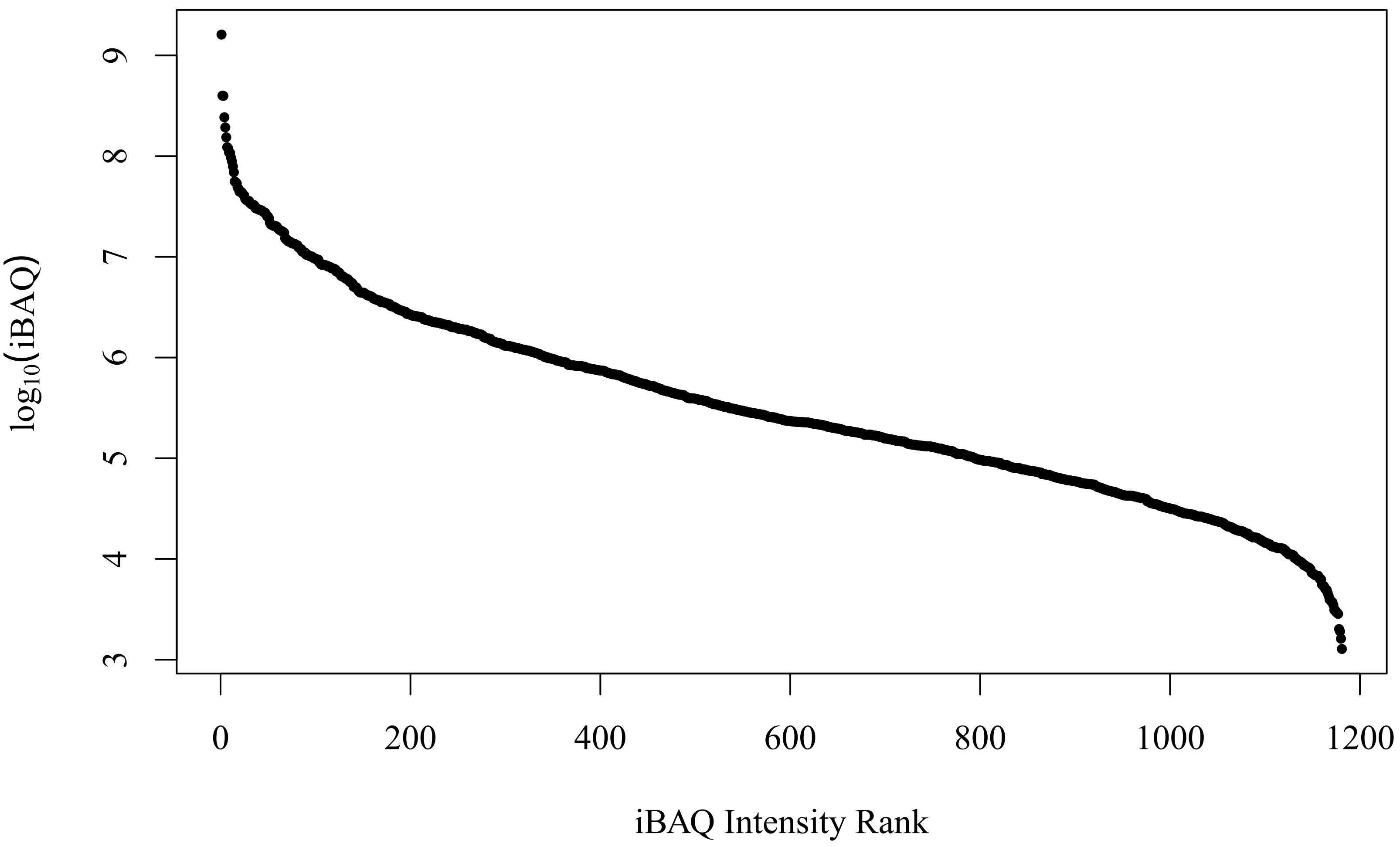

log10(iBAQ)
9
8
7
6
5
4
3
0
200
400
600
800
1000
1200
iBAQ Intensity Rank

Figure S4

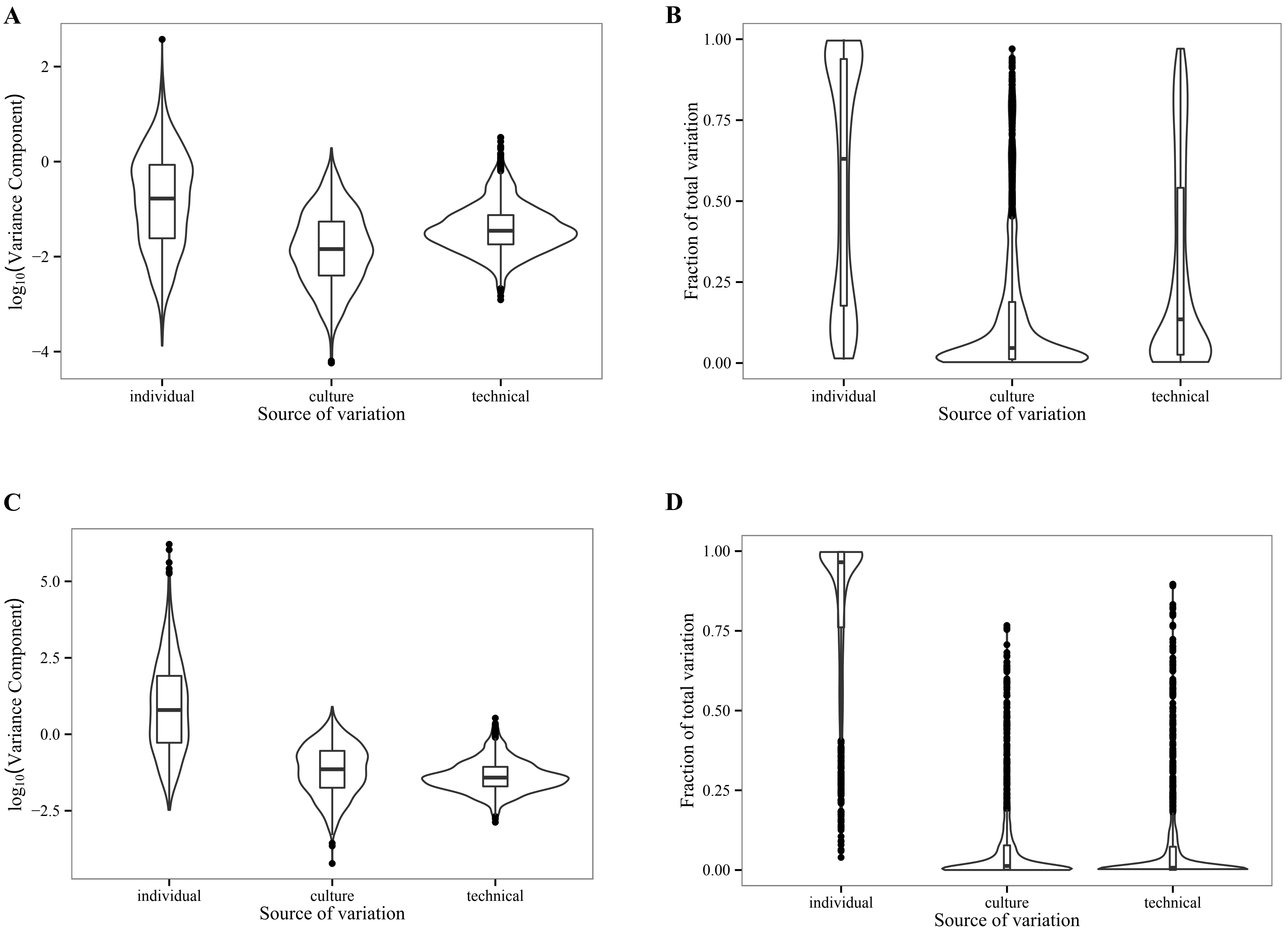
A
log10(Variance Component)
2
0
−2
−4
individual
culture
technical
Source of variation
B
Fraction of total variation
1.00
0.75
0.50
0.25
0.00
individual
culture
technical
Source of variation
C
log10(Variance Component)
5.0
2.5
0.0
−2.5
individual
culture
technical
Source of variation
D
Fraction of total variation
1.00
0.75
0.50
0.25
0.00
individual
culture
technical
Source of variation

Figure S5

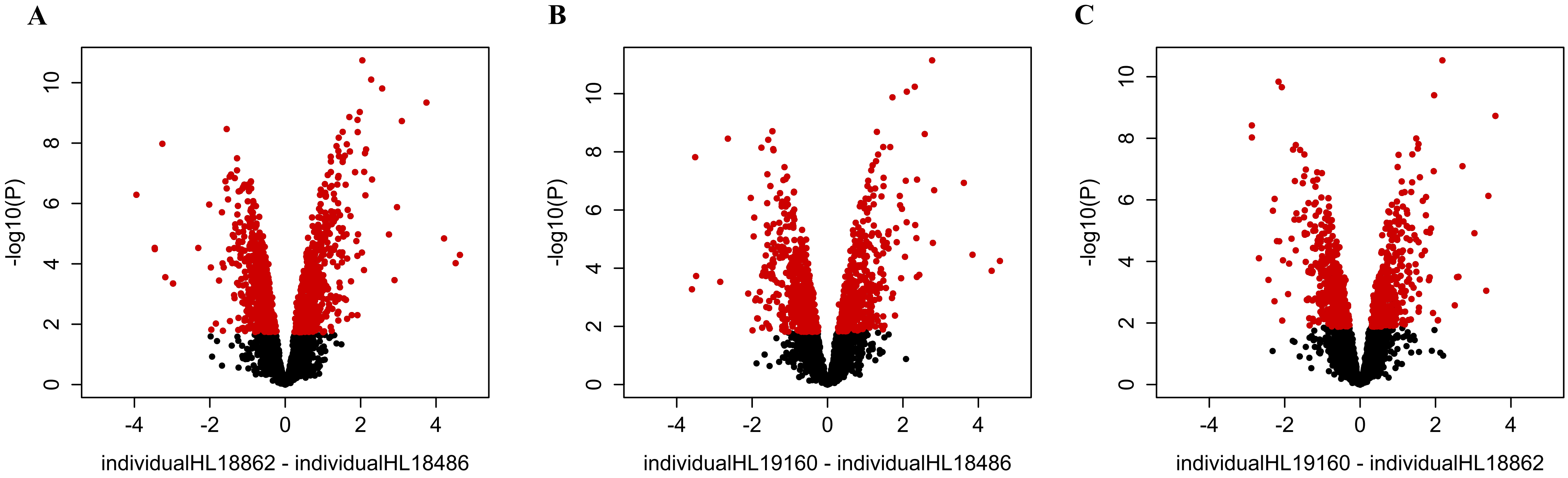
A
B
C
-log10(P)
0
2
4
6
8
10
-4
-2
0
2
4
individualHL18862 - individualHL18486
individualHL19160 - individualHL18486
individualHL19160 - individualHL18862

Figure S6

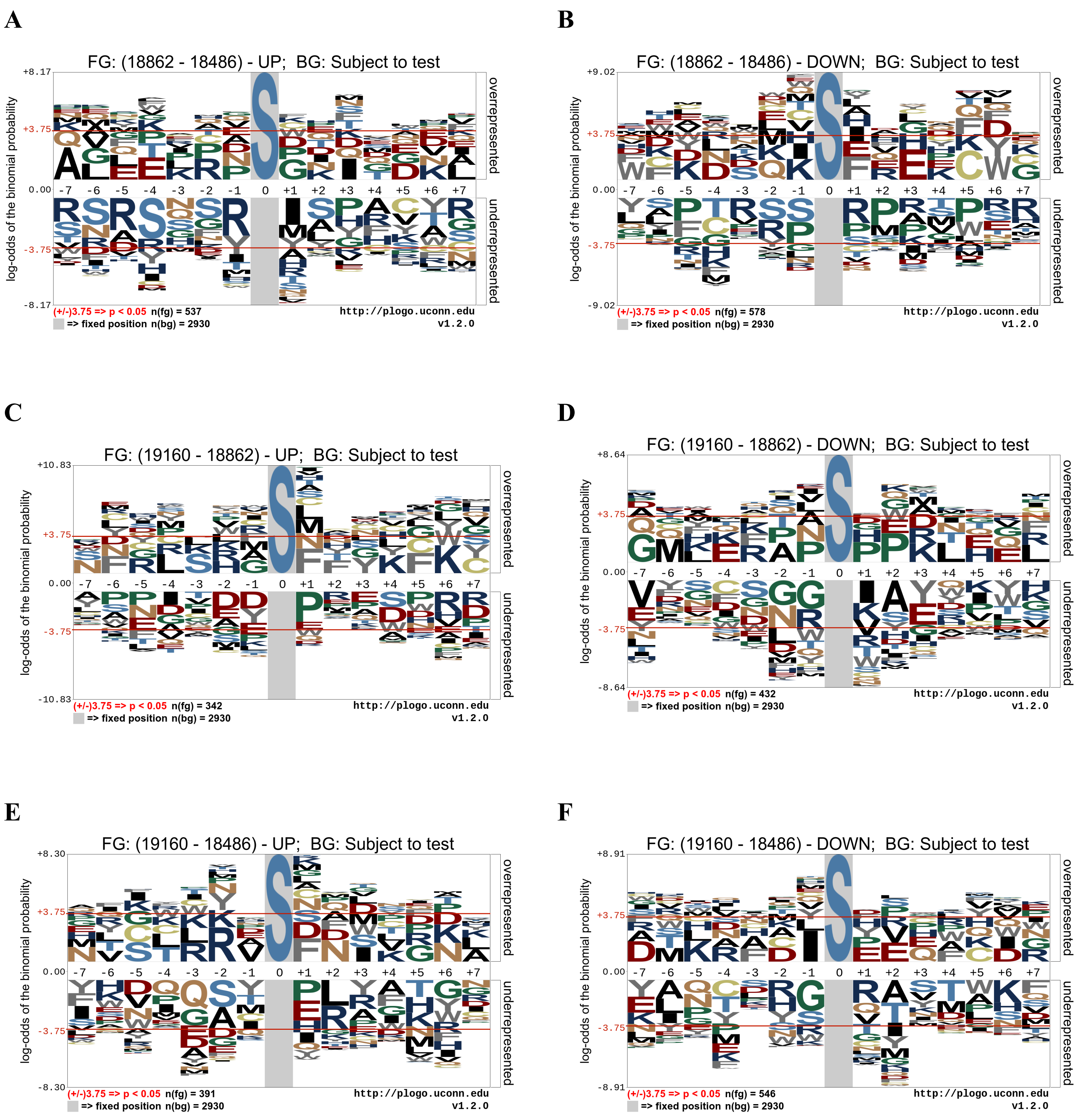
A
FG: (18862 - 18486) - UP; BG: Subject to test
(+/-)3.75 => p < 0.05 n(fg) = 537
=> fixed position n(bg) = 2930
B
FG: (18862 - 18486) - DOWN; BG: Subject to test
(+/-)3.75 => p < 0.05 n(fg) = 578
=> fixed position n(bg) = 2930
C
FG: (19160 - 18862) - UP; BG: Subject to test
(+/-)3.75 => p < 0.05 n(fg) = 342
=> fixed position n(bg) = 2930
D
FG: (19160 - 18862) - DOWN; BG: Subject to test
(+/-)3.75 => p < 0.05 n(fg) = 432
=> fixed position n(bg) = 2930
E
FG: (19160 - 18486) - UP; BG: Subject to test
(+/-)3.75 => p < 0.05 n(fg) = 391
=> fixed position n(bg) = 2930
F
FG: (19160 - 18486) - DOWN; BG: Subject to test
(+/-)3.75 => p < 0.05 n(fg) = 546
=> fixed position n(bg) = 2930
log-odds of the binomial probability
overrepresented
underrepresented
http://plogo.uconn.edu
v1.2.0

Figure S7

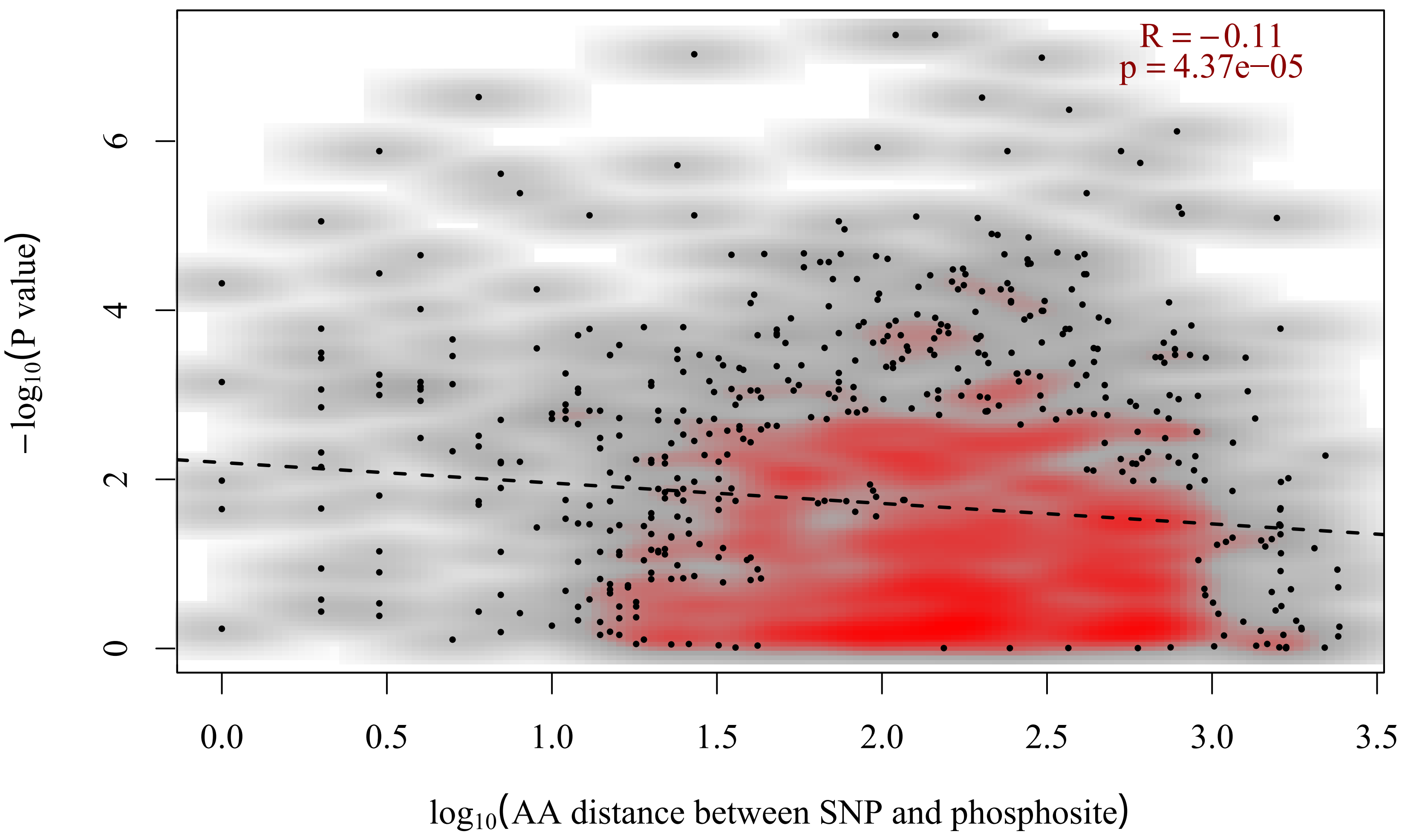

R = −0.11
p = 4.37e−05
−log10(P value)
0
2
4
6
0.0
0.5
1.0
1.5
2.0
2.5
3.0
3.5
log10(AA distance between SNP and phosphosite)

Figure S8

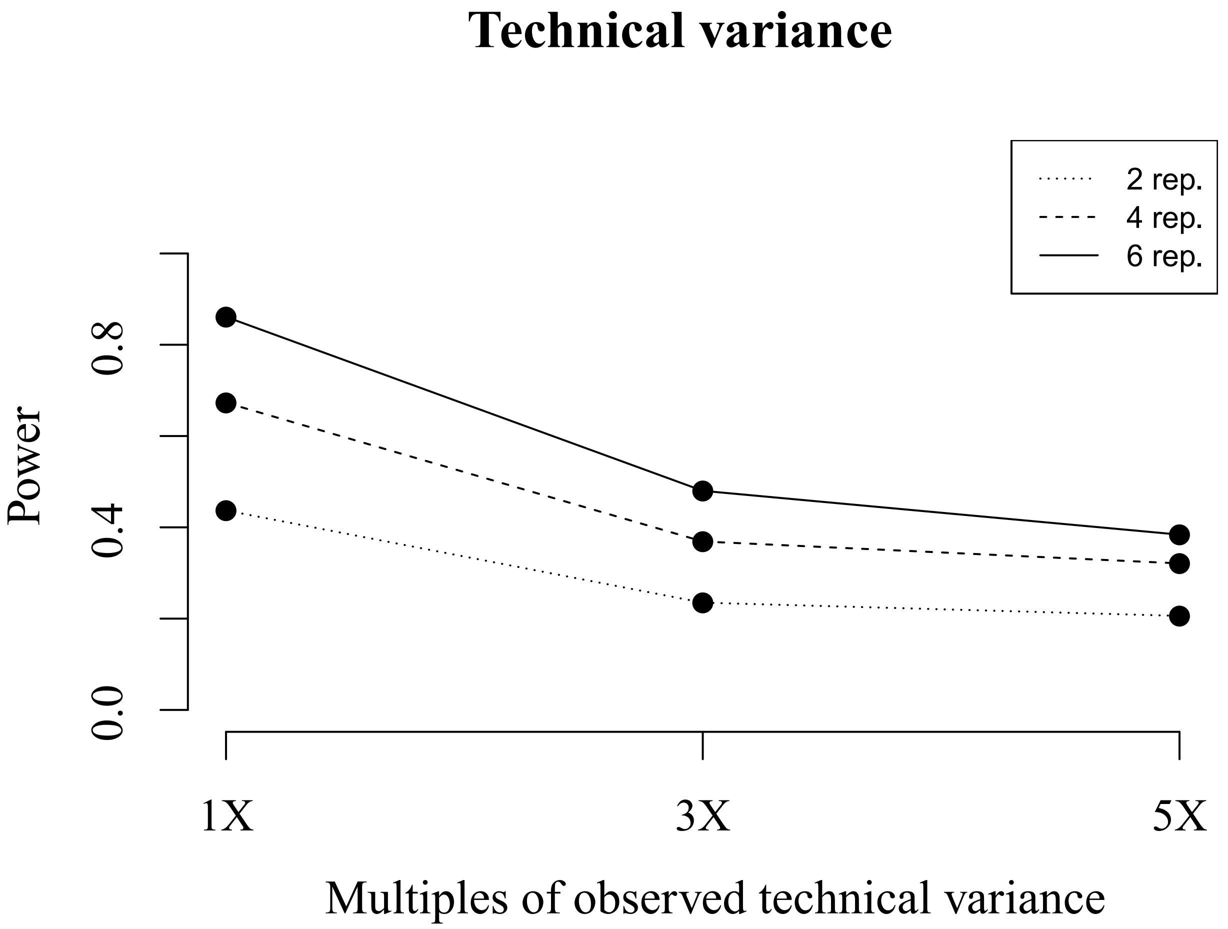
Technical variance
2 rep.
4 rep.
6 rep.
Power
0.0
0.4
0.8
1X
3X
5X
Multiples of observed technical variance
